# Supplementary material for: Seminoma and Embryonal Carcinoma Footprints Identified by Analysis of Integrated Genome-Wide Epigenetic and Expression Profiles of Germ Cell Cancer Cell Lines
Source: PLoS One. 2014 Jun 2;9(6):e98330. doi: 10.1371/journal.pone.0098330 (PMC4041891; doi:10.1371/journal.pone.0098330)
Supplement: File S1 — ZIP file containing DMRforPairs output for significant regions. Please start from the html files. (ZIP) [file pone.0098330.s008.zip › figures/10000.pdf]

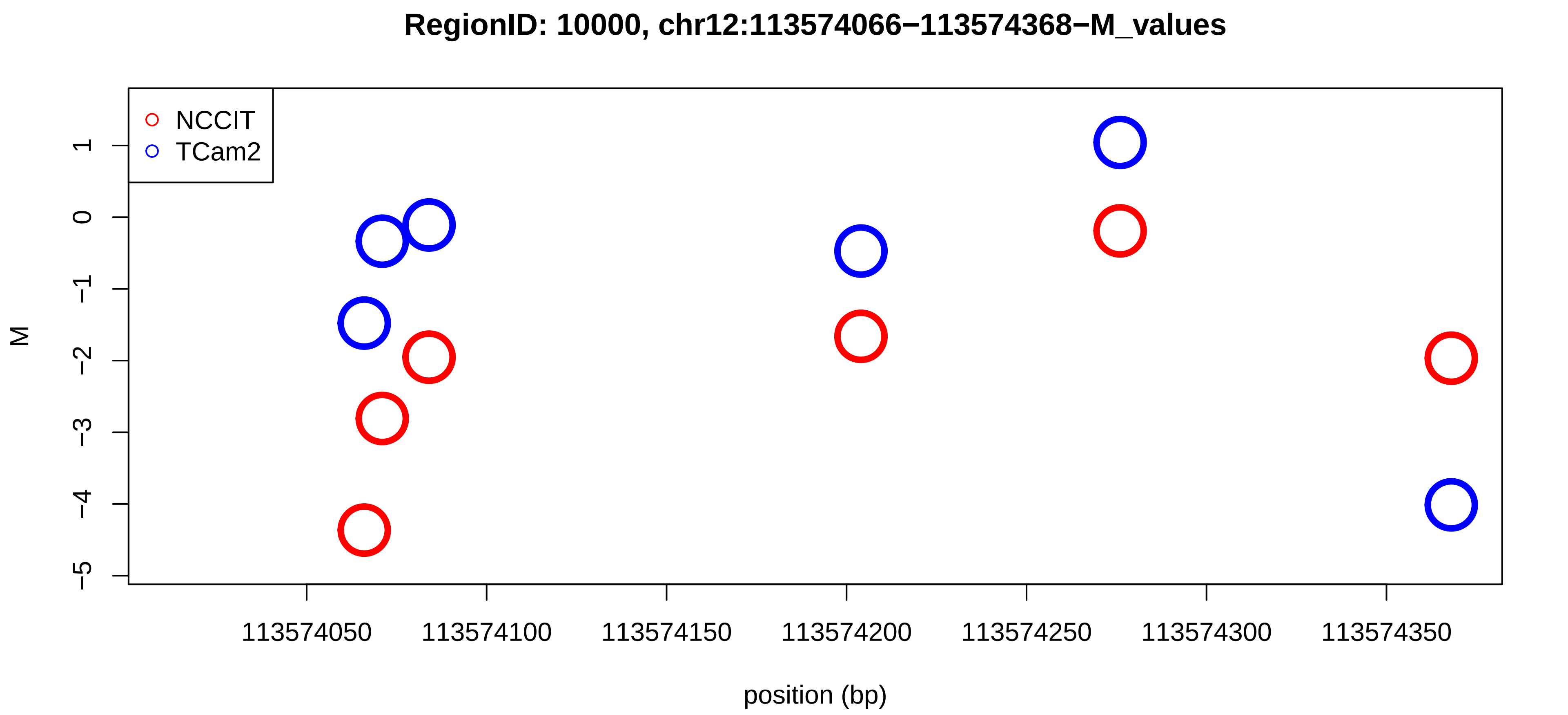

RegionID: 10000, chr12:113574066−113574368−M_values
NCCIT
TCam2
M
1
0
−1
−2
−3
−4
−5
113574050
113574100
113574150
113574200
113574250
113574300
113574350
position (bp)

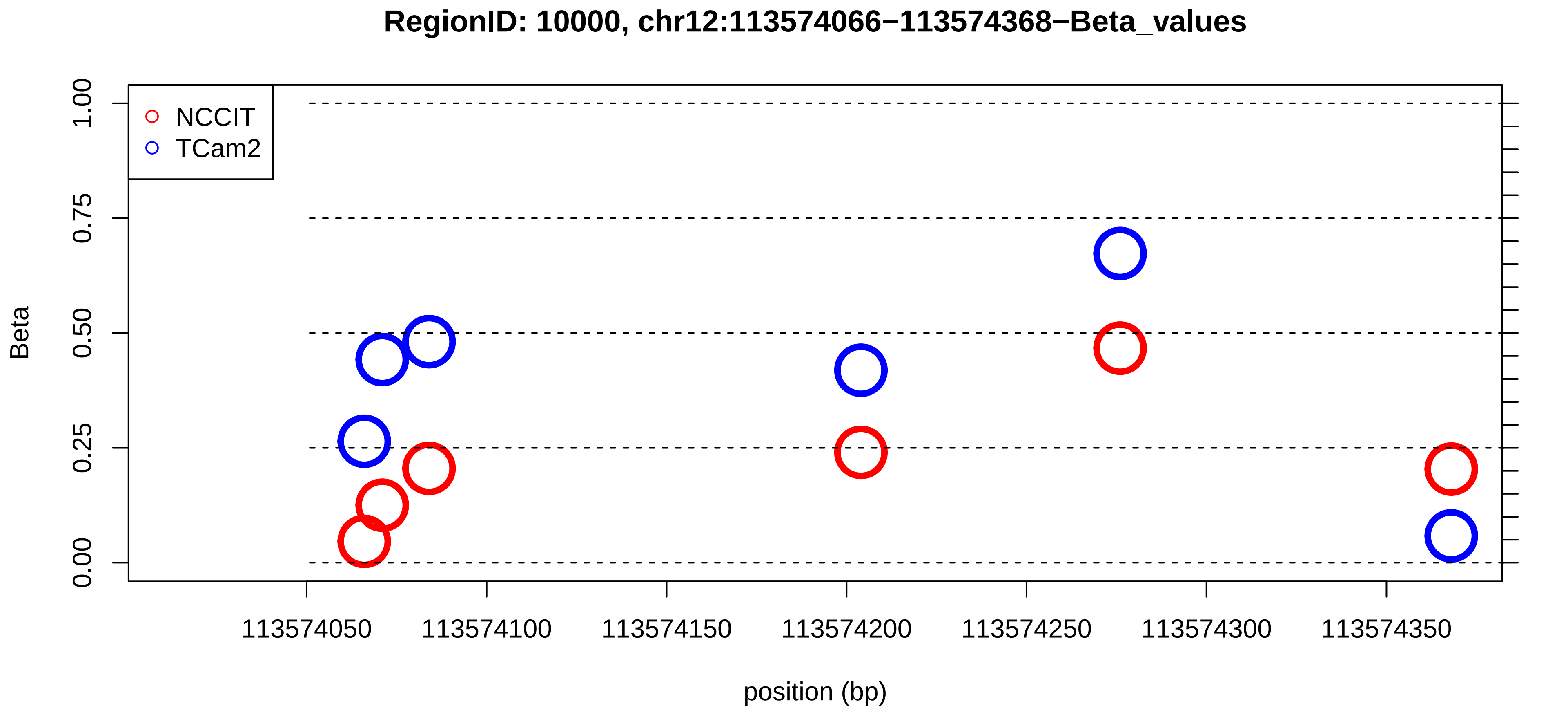

RegionID: 10000, chr12:113574066−113574368−Beta_values
NCCIT
TCam2
Beta
1.00
0.75
0.50
0.25
0.00
113574050
113574100
113574150
113574200
113574250
113574300
113574350
position (bp)
